# A Case of Asymptomatic Cement Migration Into the Pulmonary Arteries

**DOI:** 10.7759/cureus.106477

**Published:** 2026-04-05

**Authors:** Aikaterini Tzioutzia, Aikaterini Gakidi, Ioannis Beis, Ioannis Stanopoulos, Georgia Pitsiou, Afroditi Boutou

**Affiliations:** 1 Department of Respiratory Failure, G. Papanikolaou Hospital, Aristotle University of Thessaloniki, Thessaloniki, GRC

**Keywords:** cement migration, percutaneous vertebroplasty, pmma pulmonary embolization, polymethyl methacrylate, pulmonary embolism

## Abstract

Percutaneous vertebroplasty (PVP) is a minimally invasive procedure used for the treatment of pathological vertebral fractures. Polymethyl methacrylate (PMMA) is injected into the fractured vertebrae, which leaks to the surrounding tissues and travels to the pulmonary circulation in a significant proportion of cases. We report the case of a 65-year-old asymptomatic woman with multiple linear opacities on a chest X-ray, conducted two months after the procedure. The case was managed conservatively due to the absence of any concerning features. Although PMMA pulmonary artery embolism is not a rare complication, awareness remains extremely low; however, physicians should bear it in mind, as this complication may frequently be asymptomatic but, in severe cases, could lead to fatal outcomes.

## Introduction

Percutaneous vertebroplasty (PVP) and kyphoplasty (PKP) are two radiological procedures applied with an increasing frequency to treat the pain associated with the destruction of the vertebral bodies, either due to osteoporotic fractures or malignant invasion [1]. The most commonly injected material is polymethyl methacrylate (PMMA), also known as bone cement, that is inserted under fluoroscopic or computed tomography (CT) guidance [2]. It offers immediate pain relief, and in combination with the minimally invasive technique that is being used, it is constantly gaining popularity.

Alongside the expansion of its use, an increase in the reported complications is being noted, the most prominent being that of cement leakage in the perivertebral areas. PMMA alliteration outside of the vertebral body is being noted in up to 73% of cases of vertebroplasty and 33% of cases of kyphoplasty [3], the most important being that of venous intravasation in about 25% of cases [4]. Due to the fact that most of the patients with cement embolization into their pulmonary vasculature remain asymptomatic, the actual prevalence of this phenomenon remains unknown.

In this paper, we present an asymptomatic case of PMMA pulmonary embolism after PVP. This case is reported to highlight the incidental radiological detection of extensive pulmonary cement embolization in a completely asymptomatic patient, emphasizing the importance of clinical awareness, which remains extremely low, especially among non-surgeons, and the need for individualized management in the absence of clear treatment guidelines.

## Case presentation

**Figure 1:**
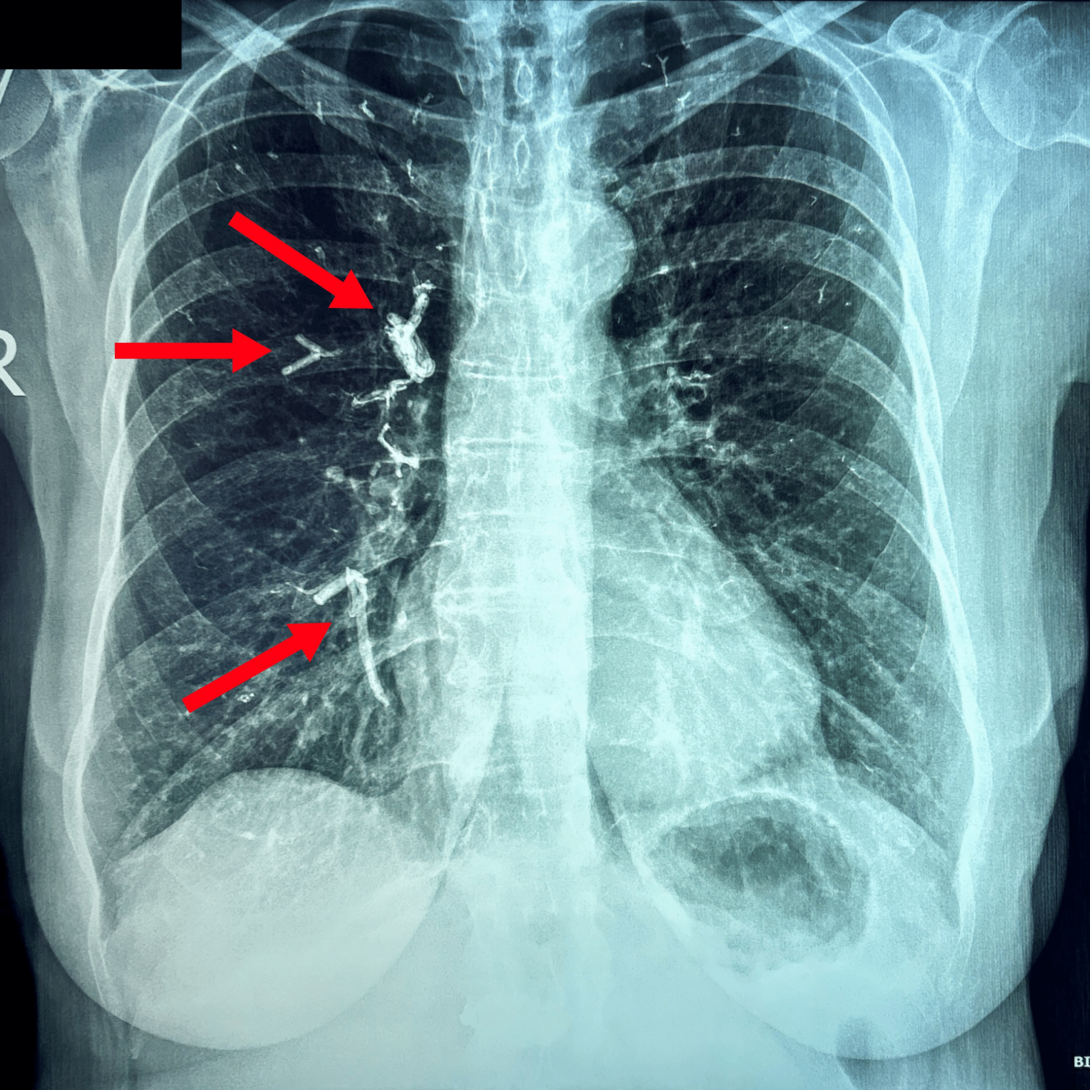
Chest X-ray of the patient. The PMMA presence in multiple pulmonary vessels is depicted with red arrows as radiopaque branching lines. PMMA: polymethyl methacrylate

We present a 65-year-old woman, a non-smoker, who visited the outpatient clinic of our hospital due to the depiction of linear opacifications on a chest X-ray (Figure 1). The X-ray was conducted as part of her annual check-up, due to the presentation of a granulomatous nodule in a previous X-ray. She reported no medical history other than the diagnosis of severe osteopenia a few months ago, with compression fractures of the L1-L2 vertebrae, for which she underwent PVP with reported alleviation of the low back pain. The procedure took place approximately two months prior to her visit to our clinic. However, no chest X-ray was conducted immediately after the procedure. The patient also denied any respiratory symptoms such as dyspnea, cough, chest pain, and hemoptysis.

**Figure 2:**
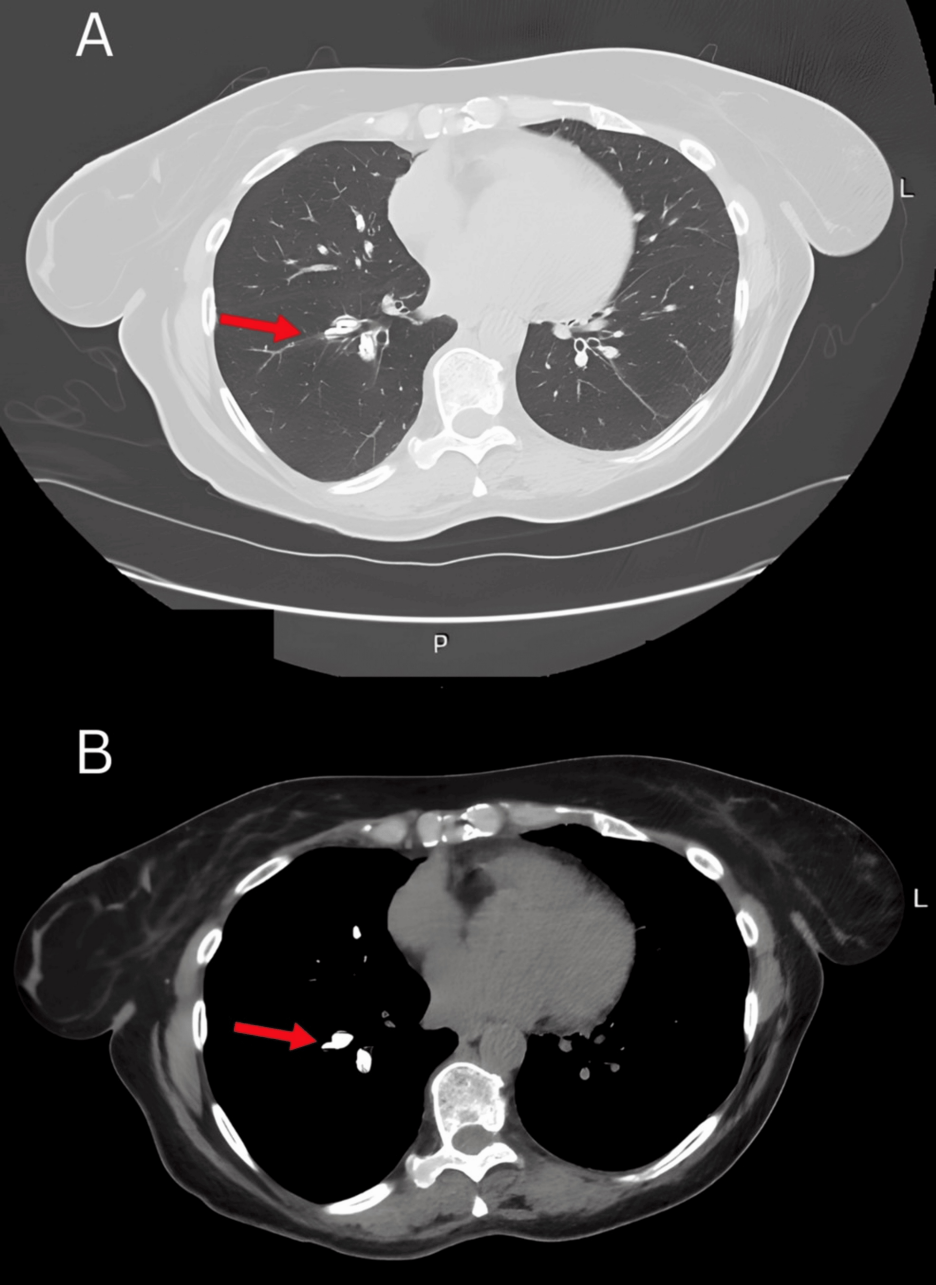
Thorax CT scan. A: lung window; B: mediastinal window projection. The PMMA presence in pulmonary vessels is depicted with red arrows as hyperdense opacities circumferential to the pulmonary vessels. PMMA: polymethyl methacrylate

Clinical examination revealed no specific pathology, and vital signs, including arterial oxygen saturation, were normal. The pulmonary function tests were within the normal range for her age, sex, and somatometric data (Table 1). She was referred for a chest CT scan for further evaluation, which demonstrated multiple linear hyperdensities along the pulmonary vessels, predominantly involving the right hilum and extending toward the lower lobe (Figure 2). The differential diagnosis included the calcification of a previous thrombi, which was excluded due to the absence of a history of pulmonary embolism, and hyperparathyroidism, ruled out based on normal calcium and parathyroid hormone serum levels.

**Table 1:** Pulmonary function test values. FVC: forced vital capacity; FEV1: forced expiratory volume in one second; DLCO: diffusion capacity for carbon dioxide; TLC: total lung capacity

Test	Best, ml	(Best/Predicted), %
FVC	2,110	62%
FEV1	1,700	65%
FEV1/FVC	-	80.3%
DLCO	-	75%
TLC	4,910	86%

Taking into consideration a previously normal chest X-ray, before the vertebroplasty procedure, a diagnosis of PMMA migration into the pulmonary vasculature was made. Due to the fact that the patient presented no symptoms, she did not receive any specific treatment, but close follow-up was arranged, and she was advised to remain vigilant for any respiratory symptoms and seek emergent help in case of appearance. The follow-up lasted for six months after the procedure, during which she did not report the development of any respiratory complaints. No repeat chest imaging was performed during follow-up due to the absence of symptoms and stable clinical status. A summary of the case is presented in Table 2.

**Table 2:** Summary of the case presentation. PMMA: polymethyl methacrylate

Patient	65-year-old, female
Main complain	Multiple linear opacities on chest X-ray (incidental, radiological detection)
Medical history	Severe osteopenia with L1–L2 compression fractures, for which she underwent percutaneous vertebroplasty approximately two months prior to presentation
Presentation	Asymptomatic (no dyspnea, cough, chest pain, hemoptysis), stable vital signs
Investigation	Chest CT: linear hyperdensities along pulmonary vessels. Pulmonary function tests: within normal limits. Arterial blood gases: normal values
Diagnosis	Pulmonary PMMA embolization
Management	Conservative with close follow- up

## Discussion

In this report, we describe a case of asymptomatic PMMA pulmonary embolization after PVP. Unfortunately, as the popularity of vertebroplasty has increased, so has the incidence of potentially lethal complications of the procedure [5]. Despite the increasing incidence of PMMA embolism, a clear algorithm for management does not yet exist [5], so awareness among physicians should be raised.

PMMA is an artificial cement that can be appropriately introduced into the vertebral marrow space. Due to its inert nature, dependable strength, and lack of toxicity, it is widely used in vertebroplasty procedures, and once in place, this artificial cement stabilizes the vertebral body, often leading to pain reduction [6]. However, it is believed that the increased intravertebral pressure after the injection of the material leads to overflow and leak of cement in the surrounding tissues [5]. The spine is vascularized by a dense valveless venous plexus that communicates through the azygous vein with the vena cava, through which the cement can reach the pulmonary circulation. Moreover, bone marrow may be displaced to the bone vessels after a traumatic procedure, causing fat microemboli to travel to the pulmonary circulation and embolize capillaries, further contributing to respiratory compromise [7]. The only risk factor that dictates immediate evaluation with CT imaging of the thorax is the visualization of leakage during the procedure [3].

Most patients remain asymptomatic, since the emboli most often occlude small peripheral lung arteries sparsely, in people with healthy lung parenchyma and appropriate respiratory reservoirs. When symptoms are being reported, those usually include dyspnea, especially on exertion, tachypnea, tachycardia, and chest pain [8]. Occurrence of persistent pulmonary hypertension has also been described in some patients [3]. Moreover, the presence of PMMA inside pulmonary vessels can initiate an inflammatory response, increasing capillary permeability and causing acute respiratory distress syndrome (ARDS) [9].

The diagnosis of cement migration and embolization of the pulmonary arteries is being made after direct visualization of the hyperdense cement inside pulmonary vessels on chest radiographs and CT scans, even without the use of contrast [7]. It is suggested that the diagnosis should be followed by an echocardiogram to evaluate for the presence of secondary pulmonary hypertension [10]. For most of the patients, gas exchange remains unaffected, since there are no structural changes of the parenchyma and the cement acts like a biologically inert agent [3].

There is no clear guideline regarding the treatment of patients with cement migration into the pulmonary vasculature. For asymptomatic patients, it is suggested that the condition may be left untreated with close clinical monitoring for symptoms of respiratory failure [11]. Symptomatic patients may benefit from a prophylactic course of low molecular weight heparin in the scope of preventing the formation of thrombi on top of the areas of PMMA deposition [3,12]. Moreover, anticoagulation therapy could also be beneficial for patients who present a higher risk of a secondary thrombus or a life-threatening complication, such as those in a hypercoagulable state due to active malignancy; thus, the decision on starting anticoagulation therapy should always be individualized. In the case we report, the lack of symptoms and any additional risk factors for thrombi formation guided the clinical decision of not initiating anticoagulation therapy.

Although anticoagulation may reduce the risk of secondary thrombi, it does not improve the increased right ventricular overload and the ventilation-perfusion mismatch caused by the cement embolization [13]. After six months of anticoagulation therapy, the cement becomes endothelialized, and it no longer poses a risk of occlusion of the vessel [11]. There are reports of patients presenting with severe respiratory and circulatory failure, requiring surgical embolectomy in order to obtain hemodynamic stability, or even wedge resection of an infarcted portion of the lung [14]. In case of acute symptoms (dyspnea, hemodynamic instability, arrhythmias) during the procedure, the procedure should be terminated immediately, and the patient should be placed in a supine position with supplemental oxygen until further treatment is initiated [1].

Since pulmonary embolization occurs in about 25% of patients undergoing stabilization of their spine using PMMA [3], it is important to remain vigilant of the symptoms, especially during the first 24 hours after the procedure. Also, a chest radiograph after the procedure can be included in the routine workup of all patients [3]. The risk of PMMA extravasation can be reduced by ensuring good technique is being used, including correct needle placement, injection of the appropriate amount of material, and high viscosity of the mixture, similar to that of toothpaste [14]. Furthermore, aspiration of the vertebral body after the placement of cement can decrease the risk of peripheral leakage [7].

## Conclusions

The expanding use of PVP and PKP for pathological vertebral fractures has led to an increase in the observed complications. Pulmonary cement embolization is a relatively common but frequently underdiagnosed complication, largely due to its often-asymptomatic clinical presentation. However, when extensive, pulmonary cement embolization may also present as an urgent or even life-threatening complication requiring emergent medical care. At present, there is no consensus regarding optimal treatment strategies, and management should be individualized based on symptom severity and the disease burden, ranging from conservative monitoring to open endarterectomy. Increased awareness of this complication is essential to ensure timely diagnosis and management in order to avoid any adverse outcomes.
